# Association of serum and aqueous humor myonectin concentrations with diabetic retinopathy

**DOI:** 10.1038/s41598-021-86677-2

**Published:** 2021-03-30

**Authors:** Huibo Sun, Zhongtao Li, Wenchao Hu, Wenjie Ma

**Affiliations:** 1 Department of Endocrinology, Qilu Hospital (Qingdao), Cheeloo College of Medicine, Shandong University, 758 Hefei Road, Shibei District, Qingdao, 266035 Shandong China; 2 Department of Endocrinology and Metabolism, Zaozhuang Municipal Hospital, Zaozhuang, China

**Keywords:** Biomarkers, Endocrinology

## Abstract

Myonectin, a newly discovered myokine, enhances fatty acid uptake in cultured adipocytes and hepatocytes and suppresses circulating levels of free fatty acids in mice. This study is performed to evaluate the association between serum and aqueous humor myonectin concentrations with diabetic retinopathy (DR). This study was performed in a population of 228 patients with type 2 diabetes (T2DM) and 72 control subjects. Diabetic patients were then divided into T2DM patients without DR, non-proliferative diabetic retinopathy (NPDR) patients, and proliferative diabetic retinopathy (PDR) patients. Serum and aqueous humor myonectin concentrations were significantly lower in the case group than in the control group. PDR patients showed significantly decreased serum and aqueous humor myonectin concentrations than in the other two T2DM patients. In addition, NPDR patients showed significantly lower serum and aqueous humor myonectin concentrations than T2DM patients without DR. Logistic regression analysis demonstrated that serum and aqueous humor myonectin was correlated with a decreased risk of T2DM and DR. Simple linear regression analysis showed that serum myonectin was negatively correlated with duration of disease, body mass index (BMI), and HbA1c. Duration of disease and BMI were still correlated with the serum myonectin after a multiple linear regression analysis. Aqueous humor myonectin was negatively correlated with duration of disease, systolic blood pressure (SBP), and diastolic blood pressure. Duration of disease and SBP was still correlated with the aqueous humor myonectin after a multiple linear regression analysis. Our investigation indicates an inverse association of serum and aqueous humor myonectin with DR.

## Introduction

Diabetic retinopathy (DR) is the main cause of vision loss in young subjects in developed areas^1^. The exact etiologic mechanism of DR, especially proliferative diabetic retinopathy (PDR) has not been elucidated. Hyperglycemia is considered as the traditional factor for DR^2^. Recent investigations focus on the role of inflammation in DR development. Many inflammatory markers have been found to be involved in the pathogenic processes of DR^3^.

Myonectin, also named as CTRP15—C1q/TNF-related protein, stimulated the fatty acid metabolism in adipocytes and hepatocytes^4^. Exercise training results in decreased myonectin level in women^5^. Myonectin inhibited inflammatory response stimulated by lipopolysaccharide in macrophages, which indicates the anti-inflammatory role of myonectin^6^. Inflammation is correlated with DR pathology. Then it is assumed that myonectin may play a protective role in DR development and progression.

Therefore, we designed this investigation to determine the correlation between serum and aqueous humor myonectin concentrations with DR.

## Materials and methods

### Study population

This study consisted of a consecutive population of 228 patients who had cataract surgery in the Department of Endocrinology or the Ophthalmology from January 2016 to December 2019. T2DM was diagnosed according to the American Diabetes Association criteria. DR was diagnosed on dilated fundus photography examination by one ophthalmologist specialized in retina who were blinded to the patients’ clinical information. DR grading was based on the results of the worst eye, and the retinopathy severity score was assigned according to the International Clinical DR Disease Severity Scale as follows^7^: (1) no retinopathy (no abnormalities); (2) non-proliferative diabetic retinopathy (NPDR): mild NPDR (microaneurysm only); moderate NPDR (more than just microaneurysms, but less than severe NPDR); severe NPDR (any of the following: more than 20 intraretinal hemorrhages in each of 4 quadrants; definite venous beading in 2+ quadrants; prominent intraretinal microvascular abnormalities in 1+ quadrant. And no signs of PDR); (3) PDR (one or more of the following: neovascularization, vitreous/preretinal hemorrhage). T2DM patients were divided as follows: T2DM patients without DR (n = 96), NPDR patients (n = 78), and PDR patients (n = 54). NPDR patients were then divided into mild NPDR (n = 34), moderate NPDR (n = 35), and severe NPDR (n = 27). Patients with intraocular surgery history, retina laser photocoagulation history, VEGF therapy history, glaucoma, other ocular disorders, and other systemic disorders were excluded. 72 healthy subjects who went on cataract surgery were considered as the control group. The subjects who had systemic disease were excluded from the control group.

This study was approved by the ethics board of Qilu Hospital (Qingdao) and all patients provided written informed consent. All experimental protocols were approved by the licensing committee of Qilu Hospital (Qingdao). All methods were carried out in accordance with relevant guidelines and regulations.

### Laboratory methods

Serum was collected from all participants during a fasting status. A paracentesis was made in the peripheral cornea next to the limbus using a 1 mL syringe, and undiluted samples of aqueous humor (0.2 mL) were obtained just before cataract surgery. Serum and aqueous humor samples were deposited into Eppendorf tubes and stored at − 80 °C until sample analyses. Finally, serum and aqueous humor myonectin concentrations were investigated using enzyme-linked immunosorbent assay method (Aviscera Biosciences, Santa Clara, CA).

### Statistical analysis

The results were displayed as means ± standard errors or interquartile range. Chi-square tests, one-way ANOVA, or Kruskal–Wallis test were performed to compare the differences between the four groups. Serum and aqueous humor myonectin differences between mild, moderate, and severe NPDR subgroups were compared using Kruskal–Wallis test. The risk factors for the presence of T2DM and DR was determined by logistic regression analysis. The correlation between serum and aqueous humor myonectin and other variables was analyzed using simple and multiple linear regression. Pearson correlation analysis was utilized to determine the association between serum and aqueous humor myonectin. Levels of statistical significance were set at *P* < 0.05.

## Results

### Clinical characteristics

As presented in Table 1, higher blood pressure, HbA1c, triglycerides (TG), as well as lower high-density lipoprotein cholesterol (HDL-C) were observed in T2DM patients than in the controls. PDR patients presented higher blood pressure compared with the controls and the other two T2DM subgroups.

**Table 1:** Various characteristics of diabetic patients and controls.

	Controls	Diabetic patients			*P*
		Without DR	NPDR	PDR	
N	72	96	78	54	
Age (years)	57.22 ± 7.14	57.89 ± 9.93	58.54 ± 10.87	56.11 ± 8.22	0.498
Gender (M/F)	37/35	45/51	42/36	24/30	0.681
Duration (years)	–	8.28 ± 1.7	10.36 ± 2.18^b^	12.06 ± 2.17^bc^	< 0.001
BMI (kg/m^2^)	25.23 ± 1.97	25.39 ± 3.74	26.21 ± 4.16^a^	25.92 ± 4.27	0.312
SBP (mmHg)	124.64 ± 9.73	134.01 ± 16.38^a^	138.78 ± 18.66^a^	148.8 ± 20.4^a^^bc^	< 0.001
DBP (mmHg)	80 ± 7.38	85.73 ± 10.78^a^	87.63 ± 11.64^a^	93.52 ± 9.94^a^^bc^	< 0.001
HbA1c (%)	5.01 ± 0.73	8.5 ± 1.72^a^	8.39 ± 1.72^a^	8.61 ± 1.8^a^	< 0.001
TG (mmol/L)	1.18 ± 0.54	1.81 ± 1.05^a^	1.73 ± 0.92^a^	1.84 ± 1.39^a^	< 0.001
TC (mmol/L)	5.2 ± 0.93	5.21 ± 1.2	5.24 ± 1.14	5.12 ± 1.01	0.937
HDL-C (mmol/L)	1.4 ± 0.29	1.23 ± 0.31^a^	1.12 ± 0.2^a^^b^	1.22 ± 0.3^a^^c^	< 0.001
LDL-C (mmol/L)	3.39 ± 0.73	3.39 ± 0.95	3.58 ± 1	3.34 ± 0.85	0.397
Serum myonectin (ng/mL)	342.04 (280.19–396.5)	307.36 (237.2–344.58)^a^	263.71 (206.16–309.23)^ab^	227.13 (179.49–258.75)^a^^bc^	< 0.001
Aqueous humor myonectin (ng/mL)	190.98 (159.62–224.69)	159.12 (131.44–183.02)^a^	128.26 (111.26–153.5)^ab^	107.15 (88.12–120.35)^a^^bc^	< 0.001

^a^*P* < 0.05 vs. control; ^b^*P* < 0.05 vs. diabetic patients without DR; ^c^*P* < 0.05 vs. NPDR patients.

*BMI* body mass index, *SBP* systolic blood pressure, *DBP* diastolic blood pressure, *TG* triglycerides, *TC* total cholesterol, *HDL-C* high-density lipoprotein cholesterol, *LDL-C* low-density lipoprotein cholesterol.

The characteristic of gender was analyzed using Chi-square tests. The characteristics of serum and aqueous humor myonectin were analyzed using Kruskal–Wallis test. The other characteristics were analyzed using one-way ANOVA.

### Myonectin concentrations

There were lower serum and aqueous humor myonectin concentrations in the three T2DM subgroups compared with healthy controls (Table 1). Decreased serum and aqueous humor myonectin concentrations were observed in PDR patients than in NPDR and T2DM without DR subgroups (Table 1). Moreover, NPDR patients presented lower serum and aqueous humor myonectin concentrations than T2DM without DR subgroup (Table 1).

**Figure 1:**
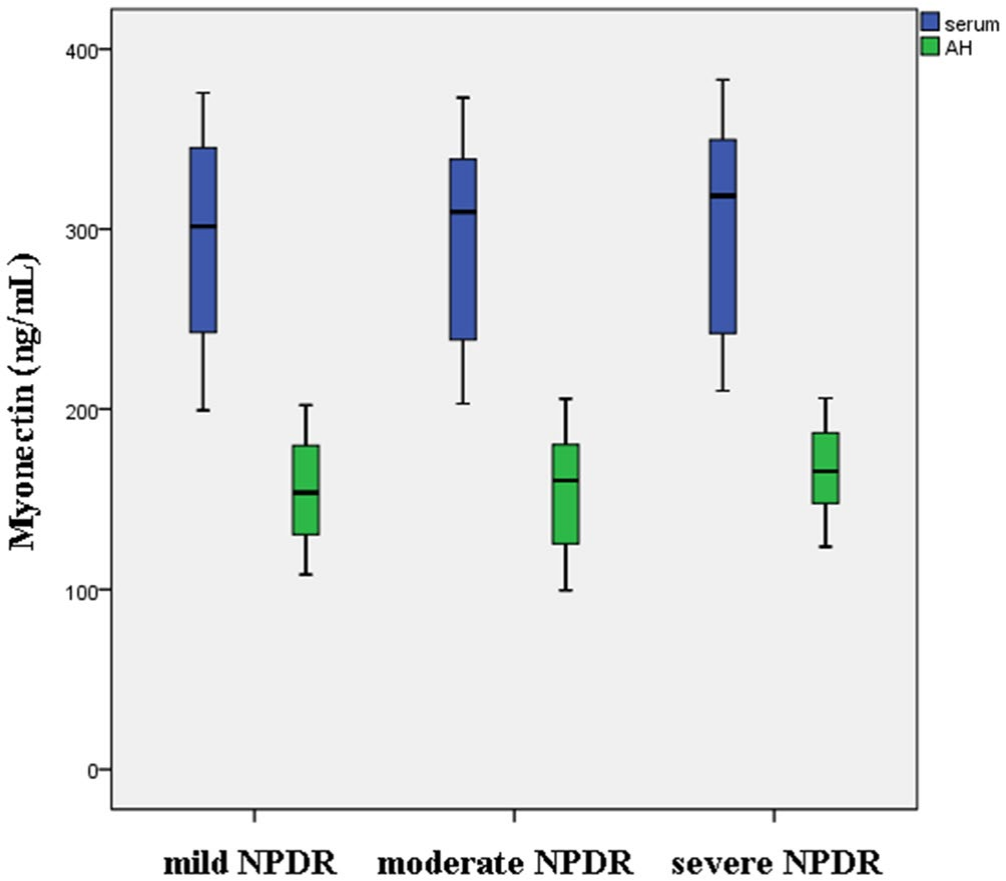
Serum and aqueous humor myonectin concentrations in mild, moderate, and severe NPDR subgroups. No difference of serum and aqueous humor myonectin concentrations were found between the three subgroups.

As shown in Fig. 1, no difference of serum and aqueous humor myonectin concentrations were found between mild, moderate, and severe NPDR subgroups.

### Association of serum and aqueous humor myonectin concentrations with T2DM

T2DM patients showed lower serum and aqueous humor myonectin concentrations compared with healthy controls [serum myonectin: 261.79 (216.96–318.81) ng/mL vs. 342.04 (280.19–396.5) ng/mL, *P* < 0.001; aqueous humor myonectin: 131.84 (111.26–162.28) ng/mL vs. 190.98 (159.62–224.69) ng/mL, *P* < 0.001]. As shown in Table 2, simple logistic regression analysis showed that systolic blood pressure (SBP), diastolic blood pressure (DBP), TG, HDL-C, serum and aqueous humor myonectin showed a trend toward an association with T2DM. Multivariate logistic regression analysis revealed that serum and aqueous humor myonectin remained a significant predictor of T2DM.

**Table 2:** Logistic regression analysis for determining the risk factors of developing T2DM.

Characteristics	Simple logistic regression		Multiple logistic regression	
	OR (95% CI)	*P*	OR (95% CI)	*P*
Age (years)	1.005 (0.977–1.035)	0.71	–	–
Gender (M/F)	1.114 (0.656–1.893)	0.689	–	–
BMI (kg/m^2^)	1.046 (0.969–1.129)	0.253	–	–
SBP (mmHg)	1.065 (1.042–1.088)	< 0.001	1.055 (1.008–1.104)	0.021
DBP (mmHg)	1.088 (1.055–1.122)	< 0.001	1.014 (0.948–1.084)	0.687
TG (mmol/L)	3.383 (1.972–5.802)	< 0.001	3.014 (1.443–6.296)	0.003
TC (mmol/L)	0.996 (0.781–1.272)	0.977	–	–
HDL (mmol/L)	0.084 (0.033–0.218)	< 0.001	0.199 (0.055–0.714)	0.013
LDL (mmol/L)	1.073 (0.795–1.447)	0.646	–	–
Serum myonectin (ng/mL)	0.983 (0.978–0.988)	< 0.001	0.99 (0.984–0.996)	0.001
Aqueous humor myonectin (ng/mL)	0.959 (0.949–0.969)	< 0.001	0.962 (0.95–0.975)	< 0.001

Abbreviation as Table 1.

Univariate logistic regression analysis was performed and the variables with a *P* < 0.05 were then entered into a multivariate logistic regression model to assess the significant independent factors associated with T2DM.

### Association of serum and aqueous humor myonectin concentrations with DR

T2DM patients with DR had lower serum and aqueous humor myonectin concentrations compared with those without DR [serum myonectin: 244.1 (199.96–282.85) ng/mL vs. 307.36 (237.2–344.58) ng/mL, *P* < 0.001; aqueous humor myonectin: 118.74 (102.71–138.91) ng/mL vs. 159.12 (131.44–183.02) ng/mL, *P* < 0.001]. As shown in Table 3, simple logistic regression analysis showed that duration of disease, SBP, DBP, serum and aqueous humor myonectin showed a trend toward an association with DR. Multivariate logistic regression analysis showed that serum and aqueous humor myonectin was still correlated with the risk of DR.

**Table 3:** Logistic regression analysis for determining the risk factors of developing DR.

Characteristics	Simple logistic regression		Multiple logistic regression	
	OR (95% CI)	*P*	OR (95% CI)	*P*
Age (years)	0.997 (0.97–1.023)	0.798	–	–
Gender (M/F)	0.882 (0.521–1.494)	0.641	–	–
Duration (years)	2.116 (1.714–2.612)	< 0.001	1.949 (1.529–2.486)	< 0.001
BMI (kg/m^2^)	1.046 (0.977–1.119)	0.194	–	–
SBP (mmHg)	1.037 (1.011–1.064)	0.005	1.012 (0.975–1.049)	0.535
DBP (mmHg)	1.041 (1.008–1.075)	0.015	1.015 (0.96–1.074)	0.598
HbA1c (%)	0.995 (0.854–1.159)	0.947	–	–
TG (mmol/L)	0.967 (0.762–1.229)	0.786	–	–
TC (mmol/L)	0.988 (0.783–1.246)	0.919	–	–
HDL (mmol/L)	0.401 (0.153–1.053)	0.064	–	–
LDL (mmol/L)	1.107 (0.835–1.466)	0.48	–	–
Serum myonectin (ng/mL)	0.983 (0.977–0.988)	< 0.001	0.986 (0.979–0.994)	< 0.001
Aqueous humor myonectin (ng/mL)	0.959 (0.948–0.97)	< 0.001	0.962 (0.948–0.976)	< 0.001

Abbreviation as Table 1.

Univariate logistic regression analysis was performed and the variables with a *P* < 0.05 were then entered into a multivariate logistic regression model to assess the significant independent factors associated with DR.

### The correlation of serum and aqueous humor myonectin with other variables

As presented in Table 4, serum myonectin was negatively correlated with duration of disease, body mass index (BMI), and HbA1c. Duration of disease and BMI were still correlated with the serum myonectin after a multiple linear regression analysis.

**Table 4:** The correlation between serum myonectin concentrations and various parameters.

Parameters	Simple regression analysis		Multiple regression analysis	
	r	*P*	β	*P*
Age (years)	− 0.026	0.693	–	–
Gender (M/F)	− 0.007	0.913	–	–
Duration (years)	− 0.325	< 0.001	− 0.287	< 0.001
BMI (kg/m^2^)	− 0.256	< 0.001	− 0.205	0.001
SBP (mmHg)	− 0.109	0.102	–	–
DBP (mmHg)	− 0.08	0.228	–	–
HbA1c (%)	− 0.16	0.016	− 0.098	0.117
TG (mmol/L)	0.054	0.417	–	–
TC (mmol/L)	0.023	0.73	–	–
HDL (mmol/L)	0.081	0.225	–	–
LDL (mmol/L)	0.003	0.968	–	–

Abbreviation as Table 1.

The correlation between serum myonectin and other parameters were analyzed using simple linear regression analysis. Then the variables with a *P* < 0.05 were entered into a multiple linear regression model to determine the contribution of various factors to serum myonectin.

Simple linear regression analysis showed that aqueous humor myonectin was negatively correlated with duration of disease, SBP and DBP (Table 5). Duration of disease and SBP were still correlated with the aqueous humor myonectin after a multiple linear regression analysis (Table 5).

**Table 5:** The correlation between aqueous humor myonectin concentrations and various parameters.

Parameters	Simple regression analysis		Multiple regression analysis	
	r	*P*	β	*P*
Age (years)	0.039	0.559	–	–
Gender (M/F)	− 0.002	0.971	–	–
Duration (years)	− 0.354	< 0.001	− 0.328	< 0.001
BMI (kg/m^2^)	− 0.057	0.393	–	–
SBP (mmHg)	− 0.238	< 0.001	− 0.209	0.044
DBP (mmHg)	− 0.188	0.004	− 0.019	0.852
HbA1c (%)	− 0.029	0.659	–	–
TG (mmol/L)	− 0.065	0.325	–	–
TC (mmol/L)	− 0.003	0.965	–	–
HDL (mmol/L)	0.011	0.873	–	–
LDL (mmol/L)	− 0.01	0.875	–	–

Abbreviation as Table 1.

The correlation between aqueous humor myonectin and other parameters were analyzed using simple linear regression analysis. Then the variables with a *P* < 0.05 were entered into a multiple linear regression model to determine the contribution of various factors to aqueous humor myonectin.

**Figure 2:**
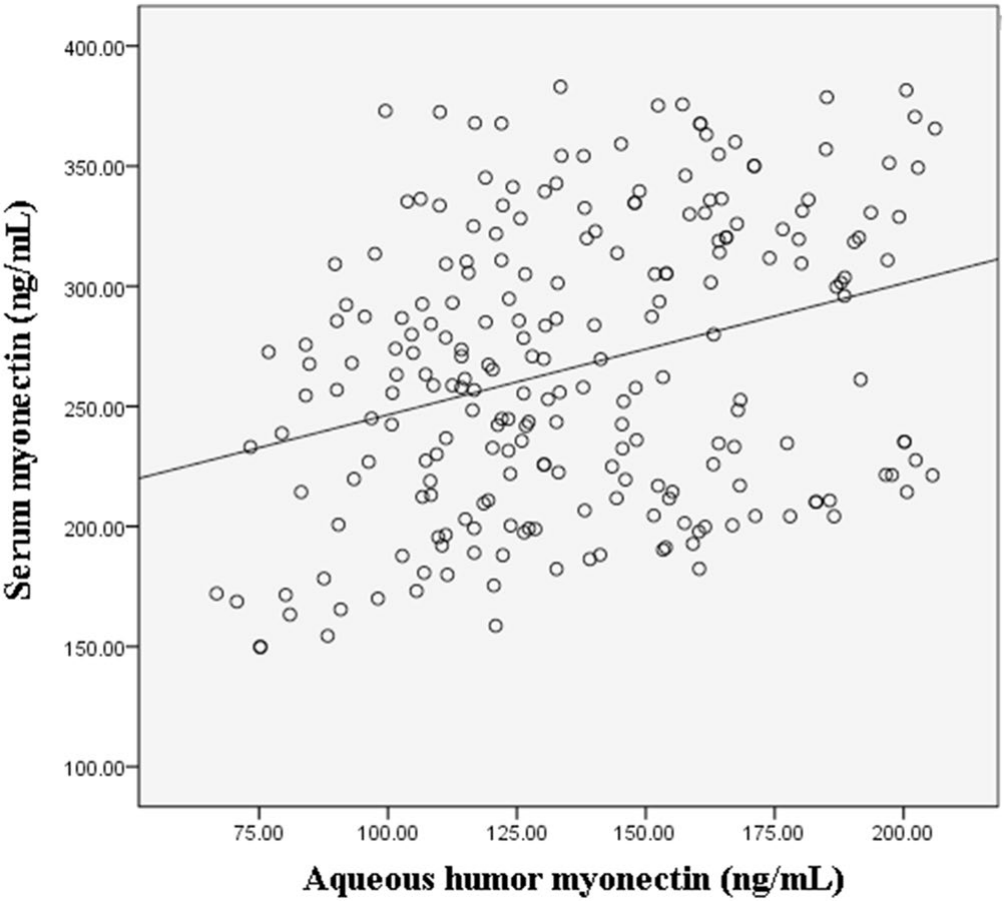
The Scatter plot showing the association between serum and aqueous humor myonectin.

Serum myonectin was correlated with aqueous humor myonectin (r = 0.308, *P* < 0.001) (Fig. 2).

## Discussion

The present study indicated that serum myonectin concentrations were decreased in T2DM patients. Zhang and Li also reported that circulating myonectin levels were significantly decreased in T2DM patients compared with the controls^8, 9^. However, another study performed in Chongqing of China showed that newly diagnosed T2DM and impaired glucose tolerance (IGT) subjects had higher circulating myonectin concentrations than normal subjects^10^. And circulating myonectin levels were higher in newly diagnosed T2DM patients than in IGT subjects^10^. These four studies are all performed in China. Therefore, the conflicting results caused by ethnic difference can be excluded. We speculate that the contradictory results may be due to different ELISA kits or population enrolled.

Myonectin is associated with macrovascular disease. Serum myonectin was higher in coronary artery disease (CAD) patients compared with controls^11^. Serum myonectin was correlated with disease severity of CAD. Zhang reported that serum myonectin levels were decreased in CAD patients compared to the non-CAD group^12^. Serum myonectin alteration may serve as a marker for CAD^12^. Myonectin-knockout mice with ischemia–reperfusion showed the enhancement of myocardial infarct size, cardiac dysfunction, apoptosis, and proinflammatory gene expression compared with wild-type mice^6^. Myonectin treatment inhibited hypoxia/reoxygenation-induced apoptosis in cultured cardiomyocytes^6^. All these results point to the close association of myonectin with macrovascular disease. It is hypothesized that serum myonectin may be correlated with macrovascular complications of diabetes. Our results demonstrated that decreased serum and aqueous humor myonectin concentrations were correlated with DR. This is the first report about the correlation between serum and aqueous humor myonectin and DR.

The potential role of inflammatory mediators in DR has been demonstrated by a variety of clinical evidence^13^. High inflammatory molecules levels have been observed in serum and vitreous sample from DR patients and animal model of diabetes^14, 15^. Recent studies demonstrated the role of myonectin in regulating inflammation. Proinflammatory gene expression were increased in myonectin-knockout mice^6^. In addition, myonectin inhibited inflammatory response stimulated by lipopolysaccharide in macrophages^6^. There was a significant relation between serum myonectin and interleukin-6, tumor necrosis factor-α in CAD patients^11^. Myonectin treatment could alleviate the anemia of inflammation induced by in mice^16^. These results indicates that myonectin is closely correlated with inflammation. Therefore, myonectin may be involved in the pathogenesis of DR by inhibiting inflammation. However, as the angiogenesis plays a key role in the mechanism of DR, further investigation may focus on the possible role of myonectin protecting from DR through the anti-angiogenic effects.

Where does the aqueous humor myonectin come from? Myonectin has been found in the eye tissue of mouse^4^. The mRNA expression of myonectin in the mouse eye tissue is about one fifth of those in the skeletal muscle tissue^4^. However, we did not know exactly which cells in the eye tissues contribute to the production of myonectin. Further study is needed to investigate the original cell in the eye to secrete the myonectin.

This study has several potential limitations. First, the conclusion is limited by relatively small sample size. Secondly, the cross-sectional nature of the data limited the strength of conclusion. The causative relation must be confirmed by future longitudinal studies. Thirdly, we did not explain the exact mechanism of myonectin protecting from DR. Further basic science study is needed to clarify the mechanism of myonectin involved in DR development or progression.

In short, serum and aqueous humor myonectin concentrations are negatively correlated with DR.
